# A novel technique to avoid intraoperative giant tear slippage: the bimanual double aspiration technique

**DOI:** 10.1186/s40942-021-00309-5

**Published:** 2021-05-27

**Authors:** Javier Placeres Daban, Daniel Artieda García, Sebastian Yaluff Portilla, José Isidro Belda Sanchís

**Affiliations:** 1 Torrevieja Hospital, Road CV 95, s/n, 03186 Torrevieja, Alicante Spain; 2 Quirón Marbella, 22, Severo Ochoa Avenue, 29603 Marbella, Málaga Spain

**Keywords:** Retinal slippage, Retinal detachment, Retinal surgery, Giant retinal tear

## Abstract

**Background:**

The aim of this paper is to present a novel bimanual double aspiration technique to avoid intraoperative giant tear slippage. The major problem of giant retinal tears (GRT) surgery is the mobility of the posterior flap (slippage), which has been classically solved by the use of intraoperative perfluorocarbon liquid (PFCL). However, avoiding slippage of the posterior flap can be a serious technical challenge when the PFCL is removed, especially when a GRT circumference is > 180°.

**Methods:**

Conventional three-port 23-gauge pars plana vitrectomy (PPV) plus chandelier was performed in three patients with giant retinal tears (GRT), using the “bimanual double aspiration technique” with non-contact wide field viewing systems. All surgeries were performed by the same surgeon.

**Results:**

None of the three cases presented with a retinal slippage after the bimanual aspiration technique.

**Discussion:**

GRT are full thickness retinal tears that extend circumferentially more than 90° of the retina. Management of GRT is a challenge for the vitreoretinal surgeons because the higher risk of proliferative vitreoretinopathy (PVR), re-detachment and increased risk of retinal slippage; this last can occur intraoperative or postoperative. Retinal slippage is not uncommon but far under-reported and can lead to various complications such as hypotony, retinal folds, and may exacerbate PVR formation. We performed bimanual double aspiration technique to avoid intraoperative giant tear slippage. We believe that this maneuver may avoid slippage by drying the posterior edge of the GRT. There were no complications related with the technique, and no additional equipment was needed.

**Conclusion:**

In summary, “bimanual double aspiration technique”, is a simple, effective, safe and economic maneuver that could be a good option to avoid intraoperative slippage in giant retinal detachment surgery, thus achieving the stabilization of the posterior retinal flap.

**Supplementary Information:**

The online version contains supplementary material available at 10.1186/s40942-021-00309-5.

## Background

GRT are full thickness retinal tears that extend more than 90 degrees circumferentially of the retina [1]. Management of GRT is challenging for the vitreoretinal surgeons: they cause a higher number of proliferative vitreoretinopathy (PVR), the retina is more prone to re-detachment and increased risk of retinal slippage; this last can happen intra or postoperatively. GRT surgery may cause mobility of the posterior flap (slippage), which can be dealt by the use of intraoperative PFCL [2, 3]. However, avoiding slippage of the posterior flap might be a serious technical challenge when the PFCL is removed, especially when GRT circumference is > 180°. To overcome this problem direct PFCL/SO exchange using SO as a primary tamponade has also been described but its difficult in manipulation together considerable amount of PFCL needed, makes this technique more complex, expensive and time-consuming [4]. SO should be avoided in the eyes in macula-on GRT with no PVR with good visual potential as well. Other studies reported a two-stage surgical procedure to treat GRT where PFCL were used as a short-term vitreous tamponade with good safety profile and low re-detachment rate [5, 6]. Nevertheless, the drawbacks of this option are the multiple surgeries needed in every patient and the PFCL-related complications including raised intraocular pressure, cataract progression, and intraocular inflammation [7, 8].

## Methods

### Surgical technique

All three patients with GRT were treated with the same technique and by the same surgeon (JPD). Local Anesthesia was induced by peribulbar nerve block. Conventional three-port 23 gauge pars plana vitrectomy (PPV) plus chandelier was performed, with non-contact wide field viewing systems. Central and exhaustive peripheral vitrectomy was performed, all the vitreous traction was removed from the GRT with retinectomy of the anterior flap. PFCL was injected slowly over the posterior pole and reached the posterior border of the retinal break, subsequently draining the subretinal fluid through the original retinal break. During the fluid-air exchange, “the bimanual double aspiration maneuver” was done; simultaneous active aspiration of the PFCL bubble near the optic nerve head and passive aspiration of the subretinal fluid on the posterior border of the retinal break was done, assisted with a backflush 23 g cannula. Swept movements of the passive aspiration at the level of the posterior retinal flap were performed, to avoid slippage of the GRT. After complete fluid- air exchange, three to four rows of endophotocoagulation were applied around the retinal break. In cases, like swelling or thickening of GRT flap, endophotocoagulation may be performed under PFCL. SO or 14% of perfluoropropane (C3F8) was used for internal tamponade at the end of surgery. No scleral buckling was needed (see Additional file 1: Video S1, which shows the “double aspiration technique”).

## Results

None of our three patients had retinal slippage, and the “bimanual double aspiration technique” was a safe and simple procedure that achieved a dried posterior edge of the GRT, avoiding slippage. No additional equipment was required to complete this technique. (See Table 1).

**Table 1:** Epidemiologic data

Patient	Age	Gender	Eye	Macular estatus	Endotamponade	Visual acuity at 12 months
1	61	Male	Right	On	Silicone oil	0.84
2	54	Female	Right	Off	Silicone oil	0.4
3	59	Male	Right	On	Gas	1

## Discussion

Avoiding retinal slippage of the GRT is a complication that vitreoretinal surgeons often have to face. There are not many intraoperative techniques described to deal with this complication, and most of them require a massive use of PFCL. We have presented three cases of GRT in which a novel maneuver (bimanual aspiration technique) succeeded in these patients.

Failure of retinal detachment surgery in eyes with GRT can be related to the inadequate tamponade to the retina by gas or SO, which can allow fluid to seep under the edge of the tear [2, 3]. Retinal Slippage is not uncommon but far under-reported and can lead to various complications such as hypotony, retinal folds, and may exacerbate PVR formation [3]. Slippage typically occurs during the fluid-air exchange due the presence of a wedge of aqueous in the interface between the air and the PFCL. As the exchange progresses this fluid at the posterior edge of the GRT, can be pushed posteriorly causing a curvilinear fold of the retina. It is then mandatory to obtain a complete drying of the posterior flap of the GRT during the exchange. However, a complete dryness of the fluid is hard to achieve in some cases. Therefore, some retinal surgeon recommends direct PFCL/SO exchange that is associate with less retinal slippage [6, 8]. This technique is challenging, and a film of fluid can remain between SO and PFCL producing the slippage of the tear.

## Conclusion

In summary, “bimanual double aspiration technique” could be a safe, effective, cheap and less time-consuming option to avoid intraoperative slippage in giant retinal detachment surgery, achieving a safe stabilization of posterior retinal flap.

## Supplementary Information

**Additional file 1: Video S1.** It shows double aspiration technique

## Declarations

### Availability of data and material

Data sharing is not applicable to this article as no datasets were generated or analyzed during the current study.
